# How is mental health associated with dental health in refugee children?

**DOI:** 10.1007/s40368-026-01188-w

**Published:** 2026-03-06

**Authors:** I. Nehring, H. Streicher, U. C. Wölfle, V. Mall, A. Hahnefeld

**Affiliations:** 1 Chair of Social Pediatrics, Technical University Munich, Munich, Germany; 2 German Center of Child and Adolescent Health, DZKJ, Munich, Germany; 3 University Hospital, LMU Munich, Munich, Germany; 4 HMU , Health and Medical University Potsdam, Potsdam, Germany; 5 Kinderzentrum München, kbo, Munich, Germany

**Keywords:** Oral health, Preschoolers, PTSD, Parental mental health

## Abstract

**Background:**

Refugee families often arrive in Europe full of hope after facing many challenges. It is known that a significant proportion of them suffer from post-traumatic stress disorder (PTSD) and that this is associated with bad health status, including oral health in adults. While it is known that refugee children have poor dental health, it remains unclear if this is related to their mental health. We aimed to find out if children’s PTSD status was associated with their dental health. We further examined if parental mental health is associated with children’s dental health.

**Methods:**

The study is part of the German INterCuLtUral Child DevelopmEnt Studies (INCLUDE). Dental and psychological examination of *n* = 44 children between 1.5 and 6 years was conducted. Dental health was assessed with dmft value and O’Leary Plaque Index (PI). The Child and Adolescent Trauma Screening was applied to test for PTSD symptoms in children. Parental mental health was assessed with the refugee health screener (RHS). Descriptive analyses and multiple linear regression were performed to test the association between child’s PTSD and dental health with parental mental health.

**Results:**

Mean dmft value was 4.98 (± 3.69), mean PI was 40.92 (± 14.76). PTSD was detected in 30% of the children. There were no statistically significant differences between children with and without PTSD symptoms pertaining dental health. Linear regression yielded child’s age and parental RHS score to be positively associated with child’s dmft value.

**Conclusions:**

Children’ had poor dental health status, regardless of the presence of PTSD. Parental mental health was associated with child dental health. Interventions and programs for refugee children should consider parental mental health as well.

## Introduction

Worldwide, the number of people who are forcibly displaced is still growing (United Nations High Commissioner for Refugees 2025). Insufficient hygienic and medical conditions both in their home countries as well as on their flights often lead to poor health status in the refugee community (Berlaer et al. 2016). Besides infections, respiratory diseases, gastrointestinal diseases and injuries, dental health is also an issue in this population (Nehring et al. 2021). Inadequate hygiene due to conditions on the move but also insufficient knowledge about the importance of regular toothbrushing may lead to increased prevalence of caries and plaque in a refugee population (Ahmad et al. 2023). In addition, threatening experiences in their countries as well as on the flights are frequent causes of mental disorders, such as PTSD. Around 20–30% of refugee children (Oleimat et al. 2023) and adults that arrived in Europe suffer from PTSD (Nehring et al. 2021). The three core symptoms are avoidance, re-experiencing, and hyperarousal (Pausch & Matten 2017). Since people affected by PTSD may neglect their own body, care and health, poor dental health might be associated with PTSD (Friedlander et al. 2004). In veterans, it was shown that combat PTSD was associated with poor dental health (Muhvić-Urek et al. 2007). A recent study showed that Egyptian preschool children with early childhood caries experienced increased cortisol levels, indicating a significant biomarker for stress (Hussein et al. 2025). It is also reported that people with PTSD suffer from myofascial and orofacial chronic pain more often than people without PTSD (de Oliveira Solis et al. 2017). A previous study has shown a significantly poorer dental and gingival health in 9–14-year-old Syrian children with PTSD compared to non-PTSD controls (Hamid et al. 2021). However, it is unclear whether this is also the case in younger children. Since toddlers and preschool children are more dependent on their parents’ care than teenagers, parental well-being should be taken into account in the context of child’s dental health. Furthermore, children with PTSD tend to show more fear and do not want to be touched, hence several studies show PTSD to be associated with fear of dental treatment (Høyvik et al. 2024; Simone et al. 2022). However, these studies included only adults and it remains unclear whether children with PTSD may also show certain anxiety behavior during dental examination.

The present study was conducted to get a deeper insight into the dental health of refugee children with and without PTSD. We aimed to answer the following research questions:

1. Do children with PTSD symptoms have different dental health compared to children without PTSD symptoms?
2. Is there an association between parental mental health and child’s dental health?
3. Do children with PTSD symptoms show different behaviors during dental examination compared to children without PTSD symptoms?

## Methods

This study is a cross-sectional sub-analysis of data from the INterCuLtUral Child DevelopmEnt Studies (INCLUDE) that focus on different aspects of the child’s development in the context of social, migration and trauma background in a refugee population. The study was conducted between June 2021 and August 2022; dental examinations took place in 2021. Details can be found elsewhere (Hahnefeld et al. 2025). Ethical approval was given by the TUM ethic commission (vote No. 221/18 S-KK) for the whole study and for the dental examination.

Children and their parents were recruited in two reception camps around Munich, Germany. Inclusion criteria were children’s age of 1.5–6 years, having a refugee background, being accompanied by at least one parent, and being accommodated in one of the participating reception camps. Children with severe developmental disorders were excluded. Eligible families were contacted in the camps and received information about the study. If they were willing to participate, the dental examination was conducted immediately afterward. All participating parents provided written consent, and all participating children provided verbal consent. A total of 50 children received dental examination.

Sociodemographic data including sex, age, country of origin, parental education and literacy were collected via questionnaires and interviews partly supported by interpreters, if English skills were not sufficient.

### Dental examination

The dental examination was conducted monocentric, by one dentist, on a normal chair, utilizing a conventional headlamp, cotton rolls for drying and using plane mouth mirrors and WHO CPI probes. To assess caries prevalence the dmft index was applied using the WHO assessment system (World Health Organization 2013). In short, for dmft each tooth is evaluated based on the criteria decayed, missing or filled. If one of these criteria is met, a point gets assigned, considering that only one point per tooth is possible. The total dmft score is then obtained by summing all points, with a maximum possible score of 20 for the primary dentition. Oral hygiene was evaluated after Mira-2-Ton (Hager & Werken) application and rinsing using the O’Leary Plaque index (O’Leary PI) (O'Leary et al. 1972). The O’Leary PI was calculated by dividing the number of plaque positive surfaces (mesial, distal, vestibular and oral) by the total number of examined surfaces and then multiplying the result by 100 to obtain a percentage. (Wolf et al. 2012). General explanations, oral hygiene instructions were done using pre-recorded audio messages and child appropriate aids (dolls, pictures) (Bundeszentrale für gesundheitliche Aufklärung, Stand 2021). Frankl children’s cooperation during the dental examination was recorded and children’s behavior was categorized as definitely positive (i.e., showing interest or laughing), positive, negative and definitely negative (i.e., impossible to complete examination) (Frankl 1962).

### Mental health

To assess the children’s mental health, parents were asked to fill out the Child and Adolescent Trauma Screening (CATS) (Sachser et al. 2017). This is a validated and publicly available screening tool to evaluate conceivable traumatic experiences and post-traumatic stress symptoms in accordance with DSM-5 Criteria for PTSD. The recommended cutoff value of ≥ 16 as an indication for a clinically relevant level of post-traumatic stress symptoms was applied.

Parental mental health was evaluated using the Refugee Health Screener (RHS-15), an empirically validated and culturally adapted screening instrument specifically designed with and for individuals with refugee background. The screening tool identifies symptoms indicative of various mental health disorders with a 14-item symptom questionnaire, resulting in an item score between 0 and 56 with a higher score indicating more symptoms (a score ≥ 12 is a positive screening result). The second part of the RHS asks the parents to show how they feel on a graphical distress thermometer (0—“things are good” to 10—“I feel as bad as I ever have”). A distress thermometer score of ≥ 5 was interpreted as a screening result indicating elevated stress (Pathways to Wellness. Integrating refugee health and well-being).

### Statistical analyses

Normal distribution was assessed using the Kolmogorov–Smirnov test. For non-parametric testing of the dmft, the Mann–Whitney *U* test was used. For the parametric distributed Plaque Index by O’Leary, the *t* test was applied. Spearman’s correlation coefficient was used to examine linear relationships between dental and mental health variables. To assess possible prediction of parental mental health on child oral health, two linear regression models with child’s age (months) and sex (1: female, 2: male), PTSD symptoms (yes/no), and parental RHS Score as predictors were calculated. Bootstrapping with 1000 replications was performed to obtain robust results, since the dmft value was not normally distributed. The outcome in model (1) was dmft value, and in model (2) PI Index. Statistical significance was set at *α* = 0.05. Statistical analyses were performed using SPSS Statistics Version 28.0.1.1. Post-hoc power analysis was conducted using G*Power (Version 3.1, University of Düsseldorf).

## Results

In total, 44 children with a mean age of 55.5 ± 14.0 months with 37 parents, mostly from Afghanistan, with full data could be analyzed. Six children were excluded due to families being transferred to other accommodations resulting in insufficient data on sociodemographic data, children’s and parental mental health (Table 1).

**Table 1:** Characteristics of study population

Children (*n *= 44)	
Mean age (months)	55,5 (± 14.0)1, range: 31–82
Sex	
Female	22 (50.0%)
Country of origin	
Afghanistan	33 (76.7%)
Palestine	2 (4.7%)
DR congo	3 (7.0%)
Venezuela	1 (2.3%)
Born in Germany^2^	4 (9.3%)
Missing	1
PTSD	13 (30.2%)
Oral health	
Number of teeth	20.27 (± 1.62)^3^, range: 16–24
Dmft	4.98 (± 3.69), range: 0–13
Decayed	4.96 (± 3.65)
Missing	0.05 (±0.21)
Filled	0 (±0)
O’Leary PI	40.922 (± 14.76); range: 9.4–61.4

Parents^1^	Mothers (*n *= 38)	Fathers (*n *= 39)
Literate		
Yes	24	25
No	12	11
Missing	2	3
Level of education		
No school visit	11	7
School, without school-leaving certificate	7	8
Secondary school	1	2
High school level	5	5
University degree	1	2
Missing	13	15
Parental RHS score	29.1 ^2^ (± 15.49)	
Parental thermometer score	6.00 ^2^ (± 3.18)	

^1^Due to siblings, the number of parents is lower than the number of children

^2^Parents were from Afghanistan, Kosovo and unknown origin

^3^Mean ± standard deviation

Mean dmft value was 4.98 (± 3.69). None of the children had any filled teeth, so the dmft value is based solely on the number of missing and decayed teeth (*d* = 4.96, *m* = 0.05, *f* = 0) (Table 1). The mean PI was 40.92% (± 14.76%). While 84% of the children showed good rapport and willingness to comply with the dentist, 16% were uncooperative and showed a negative attitude during examination.

Symptoms of PTSD were detected in 30% of the children (Table 2). Overall, children with and without PTSD symptoms did not show any significant differences regarding age, sex, dental health, and willingness to cooperate during dental examination.

**Table 2:** Comparison of children with and without PTSD symptoms

	With PTSD (*n* = 13)	Without PTSD (*n* = 31)	*p *value
Male	5 (38.5%)	17 (54.8%)	0.32
Age (months)	60.38 (± 14.36)	53.45 (± 13.42)	0.08
Number of teeth	20.08 (± 1.75)	20.35 (± 1.58)	0.31
DMFT	5.92 (± 3.80)	4.61 (3.58)	0.332
PI	43.73 (± 16.58)	39.81 (± 13.67)	0.423
Cooperation frankl			
Definitely positive	0	2	0.62
Positive	12	25	
Negative	1	4	
Definitely negative	0	0	

Parental health and well-being screening yielded a mean RHS symptom score of 29.1 (± 15.49) and a distress thermometer score of 5.99 (± 3.17). The correlation matrix (Table 3) depicts significant correlations between dmft, PI and parental RHS symptom score and distress thermometer. The linear regression model (*F* (4, 35) = 4.218, *p* = 0.007, *R*^*2*^ = 0.325) showed children’s age and parental RHS score to be significant positive predictors for dmft (Table 4). The second model with PI as outcome was not significant (*F* (4, 35) = 2.258, *p* = 0.083, *R*^*2*^ = 0.205).

**Table 3:** Correlation matrix with child’s dental and parental mental markers

	Dmft	O'Leary PI	RHS-symptom score	RHS distress thermometer
Dmft				
Spearman correlation	1	0.952	0.366	0.321
*p* value		< 0.001	0.02	0.046
N	50	40	39	50
O'Leary PI				
Spearman correlation		1	0.388	0.324
*p* value			0.013	0.044
N			40	39
RHS-symptom score				
Spearman correlation			1	0.600
*p* value				< 0.001
N				39

**Table 4:** Linear regression models with dmft and PI as outcome variable

Outcomes	dmft^1^				O ‘Leary PI			
	*F* (4, 35) = 4.218, *p* = .007, *R*^*2*^ = .325				*F* (4,35) = 2.258, *p* = .083, *R*^*2*^ = .205			
Predictor	Regression coefficient B	SE (B)	95% confidence Interval		Regression coefficient B	SE (B)	95% confidence Interval	
Sex	− 1.192	1.166	− 3.464	1.166	− 4.169	4.759	− 13.830	5.493
Age (months)	**0.123**	**0.047**	**0.036**	**0.215**	0.272	0.181	− 0.096	0.641
PTSD	− 0.662	1.454	− 3.303	2.361	− 2.561	5.445	− 13.614	8.492
RHS score	**0.081**	**0.039**	**0.003**	**0.155**	**0.348**	**0.165**	**0.014**	**0.683**

^1^Results are based on 1000 bootstrap replications

Statistically significant values are marked with **bold** font

### Post-hoc power analysis

The post-hoc power analysis revealed that with the current sample size of 44 participants (31 and 13 per group), a significance level of *α *= 0.05, and an effect size (Cohen’s d) of *d* = 0.2, the resulting statistical power was 1–β = 0.15. To achieve an adequate power level of 0.8, a sample size of 77 participants would have been required given the estimated effect size.

## Discussion

The comprehensive dental and psychological examination of 44 refugee children in a German reception camp showed no statistically significant differences pertaining to dental health markers between children with and without PTSD symptoms above threshold as rated by their parents. The linear regression model yielded children’s age and parental symptom load to be significant predictors for the child’s dmft value. Furthermore, there was no difference in behavior during the dental examination between children with and without PTSD symptoms.

The caries prevalence and the level of dental treatment (indicated by the number of filled teeth) among children from the primary country of origin, Afghanistan, are consistent with previously reported findings for this population and indicate a similar standard of oral health and care provision. Two recent studies conducted in Afghanistan found dmft values of 4.4–4.9 for children aged 6–7 years (Schwendicke et al. 2015). The level of cavity treatment, referring to latest studies, lies at 1, 8%, resembling results from this study, where we found no filled teeth at all (Lambert et al. 2024).

The dmft index was about 1 point higher in children with PTSD symptoms compared to children with less PTSD symptoms. However, this difference is not significant, which is in line with a previous study which reported no significant difference in dmft value between children with and without PTSD (Hamid & Dashash 2019). Similarly, there is no significant difference in the Plaque Index by O’Leary between the two groups, although a trend toward a higher PI in children with PTSD symptoms can be seen. In contrast, Hamid and colleagues reported a significantly higher PI in Syrian children with PTSD compared to psychologically healthy controls (Hamid & Dashash 2019). A possible explanation for the differing results is the young age of the children in our sample at which oral hygiene practices, dietary habits, and therefore, overall oral health are largely dependent on parents or caregivers. The linear regression confirms a higher parental distress to be significantly associated with a higher dmft index in the children even after adjusting for relevant confounders. There was also a medium correlation between parental stress symptoms and children’s PI. This reinforces the children’s need for parental support with personal hygiene, such as toothbrushing. However, the fine-motor skills necessary for accurate toothbrushing generally develop around the age of 8 (Berg et al. 2021). Thus, children of the present study still need their parents’ help with their (oral) hygiene. Mentally ill parents might neglect their children in this context (Davidsen et al. 2021). This suggests that children of psychologically burdened parents may receive less support and supervision in daily oral care, potentially resulting in poorer oral health outcomes and higher caries prevalence. This aligns with recent studies: a study from Japan demonstrated a negative association between maternal mental disorder (namely, postnatal depression) and child’s tooth-brushing frequency (Tsuchiya et al. 2022)*.* A positive relation between caries index (ICDAS) of school children and the depression–anxiety–stress scores of their parents was found in an Iranian study (Pezeshki et al. 2025).

Fortunately, most of the children did not show any problems during the dental examination which might be due to the voluntary character of this study. In addition, there was a nonclinical atmosphere and no dental treatment which might have caused fear or even pain.

Comparing the results of the present study with the German population reveals a similar pattern: if there is any tooth decay (i.e., dmft > 0), the dmft is very high and more than one tooth is affected. In general, the population in this study has higher mean dmft values than their German peers. This aligns with the scientific findings established by numerous Early Childhood Caries (ECC) studies, pointing out that the risk of ECC in industrialized countries primarily affects a specific population group, usually with a low socio-economic status (Senkel & Heinrich-Weltzien 2007).

### Strengths and limitations

To our knowledge, this is the first study examining the association between mental and dental health in refugee children below the age of 6 years. Children’s and parents’ mental health was assessed using validated tools. Dental examinations took place on the premises of the reception camps without a professional dental chair for positioning and thus limited ability to assess all tooth surfaces comprehensively. The absence of typical dental equipment may have led to an underestimation of caries prevalence. Language barriers may have contributed to misunderstandings, particularly regarding the assessment of early tooth loss due to severe caries. However, irregular help from interpreters, pre-recorded messages, visual aids, models, and plush toys to demonstrate procedures were applied to minimize the barriers.

The study's sample size was small, consisting of only 44 children who participated in both the dental and mental health examinations, suggesting that true differences between groups might have gone undetected. This is due to the circumstance that participants had to be staying in the reception camps for the whole time of dental and psychological examination, which was not possible for everyone due to transfers to other accommodations.

Finally, voluntary participation may have introduced bias, as parents interested in their children's health were more likely to participate. Therefore, children of less health-conscious parents might show higher caries rates and poorer oral health, which can lead to potentially underestimating the results.

## Conclusion

The results suggest that children with refugee status and their guardians are part of a risk group, regardless of the presence of child PTSD. Parental mental health seems to be very relevant for children’s dental health; thus, early oral health education and nutritional guidance should be provided in initial reception centers (AnkER–Zentren) to parents and children, for example, through targeted workshops. In addition, adequate treatment of existing carious lesions must be prioritized to ensure proper dental care.

## Data Availability

Anonymous individual participant data can be made available, in addition to study protocols, the statistical analysis plan, and the informed consent form. The data will be made available upon publication to researchers who provide a methodologically sound proposal for use in achieving the goals of the approved proposal.
